# Circadian Preference Modulates the Neural Substrate of Conflict Processing across the Day

**DOI:** 10.1371/journal.pone.0029658

**Published:** 2012-01-04

**Authors:** Christina Schmidt, Philippe Peigneux, Yves Leclercq, Virginie Sterpenich, Gilles Vandewalle, Christophe Phillips, Pierre Berthomier, Christian Berthomier, Gilberte Tinguely, Steffen Gais, Manuel Schabus, Martin Desseilles, Thanh Dang-Vu, Eric Salmon, Christian Degueldre, Evelyne Balteau, André Luxen, Christian Cajochen, Pierre Maquet, Fabienne Collette

**Affiliations:** 1 Cyclotron Research Centre, University of Liège, Liège, Belgium; 2 Cognitive and Behavioral Neuroscience Centre, University of Liège, Liège, Belgium; 3 PHYSIP SA, Paris, France; 4 Centre for Chronobiology, Psychiatric Hospital of the University of Basel, Basel, Switzerland; 5 Neuropsychology and Functional Neuroimaging Research Unit (UR2NF), Université Libre de Bruxelles, Brussels, Belgium; Vanderbilt University, United States of America

## Abstract

Human morning and evening chronotypes differ in their preferred timing for sleep and wakefulness, as well as in optimal daytime periods to cope with cognitive challenges. Recent evidence suggests that these preferences are not a simple by-product of socio-professional timing constraints, but can be driven by inter-individual differences in the expression of circadian and homeostatic sleep-wake promoting signals. Chronotypes thus constitute a unique tool to access the interplay between those processes under normally entrained day-night conditions, and to investigate how they impinge onto higher cognitive control processes. Using functional magnetic resonance imaging (fMRI), we assessed the influence of chronotype and time-of-day on conflict processing-related cerebral activity throughout a normal waking day. Sixteen morning and 15 evening types were recorded at two individually adapted time points (1.5 versus 10.5 hours spent awake) while performing the Stroop paradigm. Results show that interference-related hemodynamic responses are maintained or even increased in evening types from the subjective morning to the subjective evening in a set of brain areas playing a pivotal role in successful inhibitory functioning, whereas they decreased in morning types under the same conditions. Furthermore, during the evening hours, activity in a posterior hypothalamic region putatively involved in sleep-wake regulation correlated in a chronotype-specific manner with slow wave activity at the beginning of the night, an index of accumulated homeostatic sleep pressure. These results shed light into the cerebral mechanisms underlying inter-individual differences of higher-order cognitive state maintenance under normally entrained day-night conditions.

## Introduction

Morning and evening types differ in preferred sleep-wake times, phase of circadian rhythm markers (e.g. melatonin and core body temperature), dynamics of homeostatic sleep pressure, and time of day for optimal cognitive performance [1], [2], [3]. Thus, individual timing preferences of sleep-wake cycles and cognitive performance may come from a differential interaction of circadian and homeostatic processes. We recently tested this hypothesis in extreme chronotypes [4]. Optimal sustained attention in the subjective evening hours (high sleep pressure) was associated with more brain activity in evening than in morning types in a region of the brainstem compatible with the locus coeruleus and the anterior hypothalamus, putatively encompassing the suprachiasmatic area. Both represent key elements for the generation of the circadian wake-promoting signal [5]. Furthermore, task-related activity in the SCA decreased with accumulating homeostatic sleep pressure, which was reminiscent to the reported reduction of suprachiasmatic nuclei activity with increased homeostatic sleep pressure in mice [6], [7]. These results provide first evidence that circadian wake promotion and homeostatic sleep pressure interact within the hypothalamus to modulate human cognitive performance.

**Figure 1:**
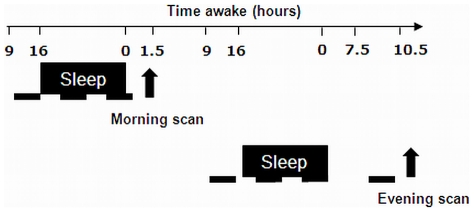
Overview of the experimental design. Subjects came to the lab 7 h before scheduled sleep time and stayed for 2 consecutive nights monitored via polysomnography (black box). They stayed under controlled light (<10 lux for wake periods and ≈0 lux for sleep periods) conditions and body posture (dashed lines). During wakefulness, sleepiness and vigilance measures were collected at hourly intervals as well as saliva samples (for melatonin assay). One and a half (morning session) and 10.5 (evening session) hours after scheduled wake up time subjects underwent an fMRI session while performing a Stroop task. Order of morning and evening sessions was counterbalanced between groups and subjects.

Besides arousal and basic forms of attention, performance in higher cognitive domains are also affected by the circadian system and sleep-wake homeostat, depending on task complexity [8]. Thus, the next question is whether those sleep-wake regulatory processes account for differences in more demanding cognitive processing and their neuronal correlates. Cognitive interference is crucial for maintaining a coherent stream of thought and thus represents a cognitive aspect required for behaving suitably in many daily live functions [9], [10]. However, how such cognitive processing and its cerebral correlates are modulated by circadian and homeostatic sleep-wake processes under normally entrained day-night conditions remains unknown. To address this question, we assessed the neural bases of performance maintenance in chronotypes with the Stroop paradigm [11], which challenges continuous control over conflicting information. Extreme morning and evening types underwent fMRI at two individually adapted time points within a normal waking day, when homeostatic sleep pressure and circadian alertness levels are low (morning session) and high (evening session; Figure 1). Ultimately, we aimed at linking these outputs to specific sleep homeostatic and circadian markers to understand the processes underlying the diurnal modulation of cognitive interference. We hypothesized that chronotypes can predict daily fluctuations in interference-related cortical responses, through a differential expression of subcortical driven wake-promoting signals throughout a normal waking day.

## Results

Part of the experimental protocol is detailed elsewhere [4]. Only essential information related to the present study is presented here.

### Population

Demographic data for all participants (16 morning and 15 evening types) are provided in Table S1. As expected, morningness and eveningness differed between the groups, as indexed by two chronotype questionnaires [12], [13].

### Timing of sleep and circadian phase markers

**Figure 2:**
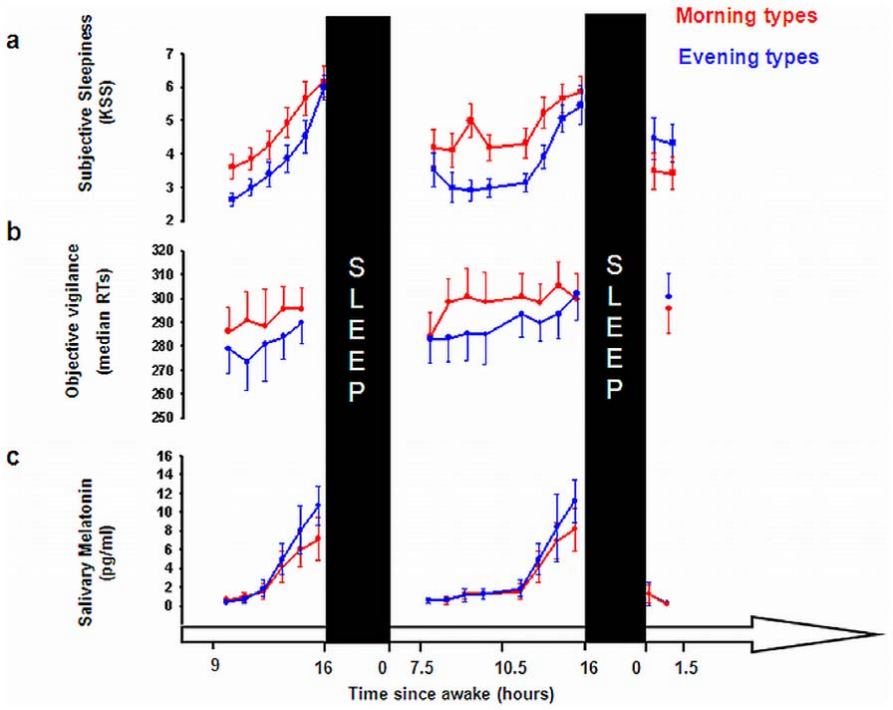
Time course (±SEM) of (a) subjective sleepiness (KSS), (b) objective vigilance (overall median RTs on PVT) and (c) salivary melatonin in morning (red) and evening (blue) chronotypes plotted according to time spent awake. One and a half (morning session) and 10.5 (evening session) hours after scheduled wake up time subjects underwent an fMRI session. Black bars indicate scheduled sleep.

Sleep and wake times were significantly earlier in morning than in evening chronotypes (t(29) = −16.26; p<0.001; Table S1). Similarly, circadian phase, as indexed by mid-range crossing (MRC) times [14] of salivary melatonin samples while awake, were significantly earlier in morning than in evening types (t(29) = −12.61; p<0.001; Table S1, Figure 2). Conversely, the phase angle between circadian phase and the sleep episode was similar in morning and evening type subjects, suggesting similar entrainment to the adopted sleep-wake cycle in both groups (t(29) = 1.28; p > 0.1; Table S1).

### Electrophysiological markers of sleep homeostasis

Polysomnographical recordings were taken during nocturnal sleep prior to fMRI sessions (data with poor recording quality were excluded; nights prior scan session: 16 for morning and 13 for evening types). All visually and automatically [15] scored sleep parameters did not significantly differ between chronotypes (Table S2).

**Figure 3:**
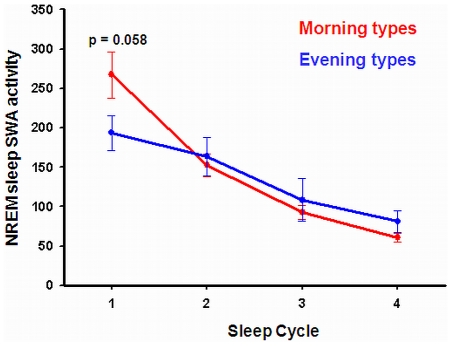
Time course of mean SWA spectral power values expressed in µV^2^ [by 0.5 Hz bin] over the first 4 NREM-REM sleep cycles (Stage 2–4), averaged over the two experimental nights for morning (red) and evening (blue) type participants.

Frontal EEG slow-wave activity (SWA; 1–4 Hz) during NREM sleep, a reliable functional index of homeostatic sleep pressure [16], [17], was computed to explore whether chronotypes differed in the dynamics of homeostatic sleep pressure. Repeated measure ANOVA on frontal SWA across four sleep cycles as within-subjects factor and chronotype as between-subjects factor yielded a significant group-by-cycle interaction (F(3,79) = 6.9; p<0.05) (Figure 3). Morning types had more SWA than evening types during the first sleep cycle (t(25) = 2.0, p = 0.058), suggesting higher homeostatic sleep pressure in morning types.

### Time course of subjective sleepiness (KSS) and objective vigilance (PVT)

Significant differences for subjective sleepiness (KSS [18]) and objective vigilance (PVT [19]) were elicited in the subjective evening hours (Figure 2), such that morning types had higher subjective sleepiness before the evening fMRI session (Mann-Whitney, U = 66.5; z = −2.1; p<0.05). Likewise, evening types tended to present higher objective vigilance than morning types before the evening scan session (data not available for one subject; t(28) = −1.89, p = 0.069),

### Interference effects: behavior

The color-word Stroop task consists in a classical interference paradigm investigating the ability for processing conflicting information [11]. In this task, subjects are required to indicate as quickly as possible the color in which a word was displayed while ignoring its meaning. Comparisons were performed between incongruent items (I; e.g. word “blue” printed in red) and congruent items (C; e.g. word “blue” printed in blue) [20] (see Methods S1 for details). Repeated measure ANOVA with “Session” (1.5 h vs 10.5 h after wake) and “Item” (I vs. C) as within-subjects factors and “Chronotype” (Morning vs Evening) as between-subjects factor was performed on accuracy and reaction time measures.

*Accuracy* analysis revealed a main effect of “Item” (F (1,29) = 11.5; p<0.005), with higher accuracy for congruent than incongruent trials (see Table 1). No significant main or interaction effects were observed for the factors “Chronotype” and “Session”. Not all volunteers produced errors during the Stroop task under these testing conditions and overall mean error rate was very low in those subjects producing errors (see Methods S1).

**Table 1:** Overall reaction times (ms) and proportion of correct responses in the different trial types of the Stroop task according to subjective time of day and chronotype.

	Morning session		Evening session	
	*Morning types*	*Evening types*	*Morning types*	*Evening types*
**Reaction times (ms)**				
*Incongruent*	947,1±142,1	878,3±161,6	977,0±134,1	865,1±214,8
*Congruent*	780,7±94,9	742,8±100,8	808,2±104,2	744,5±169,5
**Accuracy (in %)**				
*Incongruent*	97,7±2,4	96,6±1,9	96,4±3,9	95,8±3,5
*Congruent*	98,5±2,2	98,4±1,9	98,7±2,2	97,9±1,8

Analyses of *reaction times* for correct responses yielded a main effect of “Item” (Figure S1; Table 1), such that incongruent items (I) elicited significantly longer RTs than congruent trials (F(1,29) = 104.8; p<0.00001). This interference effect was not significantly modulated by time of day (F(1,29) = 0.10; p > 0.5), whereas there was a trend for morning types to perform slower as compared to evening types (F(1,29) = 3.1; p = 0.09). The interference-related interaction effect between chronotype and session did not reach significance. Separate ANOVA's on evening and morning sessions revealed no significant main effect of chronotype on Stroop interference, neither for accuracy, nor for reaction times.

### Interference effect: brain responses across a normal waking day

**Figure 4:**
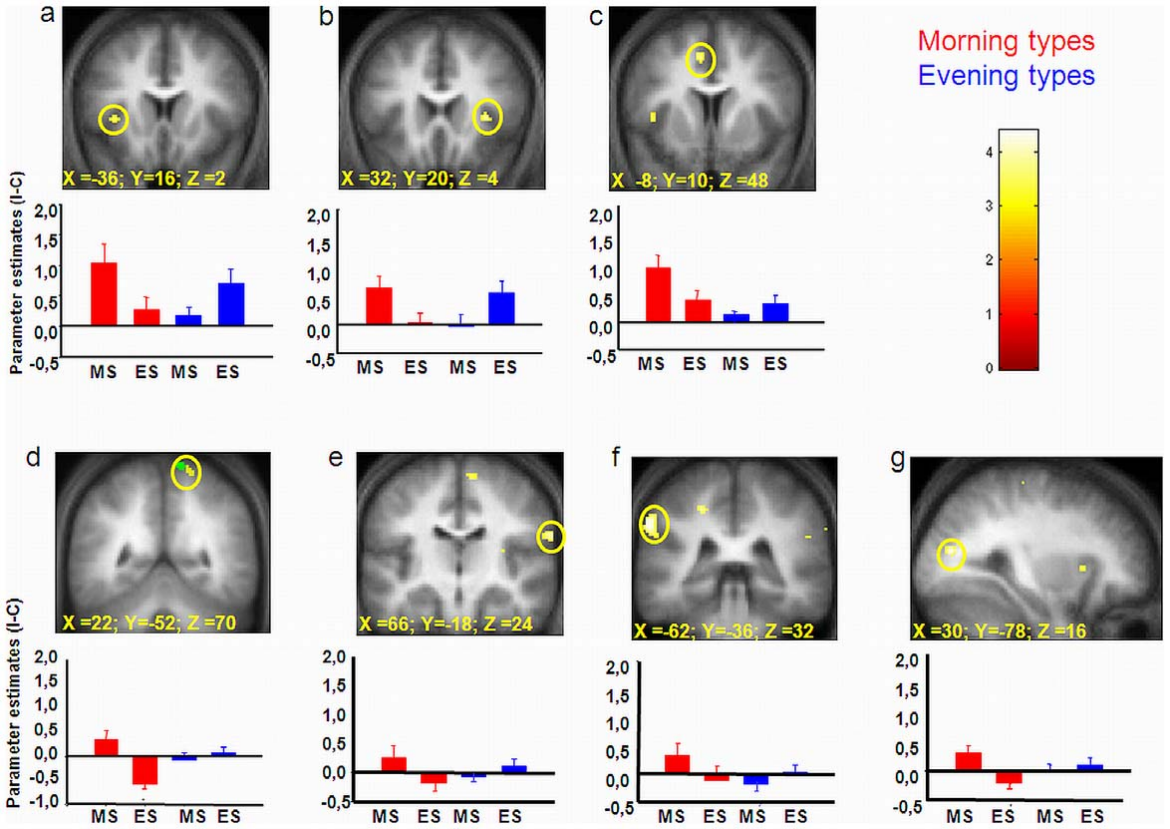
Task-related responses (I vs C) according to time of day (MS: morning session vs. ES: evening session) and chronotype (red: morning types; blue: evening types). Displays show areas (highlighted in yellow) in which activity is associated with a task-related interaction effect between Chronotype and Session [(I vs. C)*(morning vs. evening session)*(morning vs. evening types)]. Corresponding parameter estimates (arbitrary units ± SEM) are displayed. Functional results are displayed at p<0.001, uncorrected threshold, over the mean normalized structural MR image of the population.

First, we probed the neural bases of the interference effect by contrasting blood oxygen level-dependent (BOLD) responses associated with congruent vs. incongruent trials [20]. In line with prior reports [21], the interference effect (I > C) was associated with increased activity in the lateral prefrontal cortex (LPFC), anterior insula, anterior cingulate cortex (ACC) and parietal lobe (see Table S3; see Table S4 for the main effects of chronotype and time of day). Whole brain analysis revealed that task-related responses across the day evolved differently between chronotypes (Table 2, Figure 4).

**Table 2:** Brain regions showing an interaction effect between Chronotype and Session during cognitive interference in the Stroop task.

Brain regions showing an interaction effect between Chronotype and Session during cognitive interference (I>C).					
*Brain regions*	*Side*	*Coordinates*	*Z-score*	*psvc*	*Coordinates found in*
(Incongruent-Congruent)*(Morning Session-Evening Session)*(Morning types-Evening types)					
Cingulate sulcus	L	−8 10 48	3.31	0.025	[21]
		−18 −32 46	3.26	0.043	[21]
Insula	R	32 20 4	3.3	0.026	[30]
	L	−36 16 2	3.13	0.04	[21]
		−42 8 4	3.15	0.046	[21]
Superior parietal gyrus	R	22 −52 70	3.44	0.039	[47]
Inferior parietal gyrus	R	66 −18 24	3.41	0.019	[20]
	L	−62 −36 32	3.69	0.008	
Middle occipital gyrus	R	30 −78 16	3.79	0.019	[48]

Significant brain activations after correction over a small volume of interest (svc; 10 mm radius) according to structures of interest (right column).

Given the absence of behavioral differences, these disparities were not confounded by changes in performance between groups and time of day. As depicted in Figure 4, BOLD responses induced by interference (I > C) in cingulate and insular cortices decreased from the morning to the evening sessions in morning type individuals, whereas they increased or remained stable from the morning to the evening in evening types (Figure 4a–c; Table 2). Similar changes in activity patterns were observed in more posterior brain regions (e.g. superior and inferior parietal and middle occipital gyri; Figure 4d–g).

### The relationship between interference-related BOLD responses and homeostatic sleep pressure differs between chronotypes

**Figure 5:**
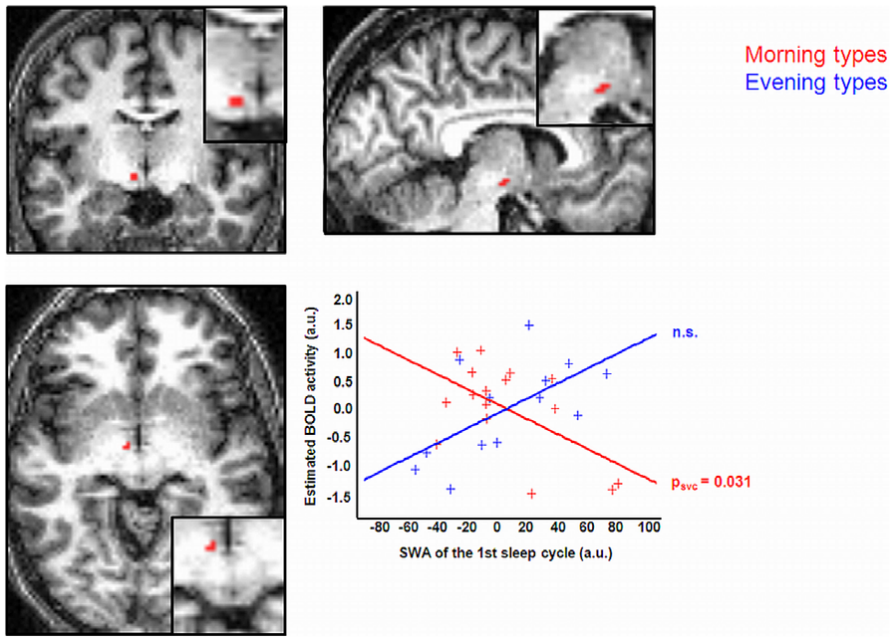
Regression analysis showing the relation between BOLD responses during cognitive interference (I > C; centered values) in the posterior hypothalamic region (sagittal, coronal and axial planes) and the amount of SWA (centered values) during the first sleep cycle according to the specific chronotype.

To explain these differences in brain responses between chronotypes, we assessed whether accumulated sleep pressure during the evening hours modulated interference-related cerebral responses, and whether this effect differed between chronotypes. We thus regressed interference-related responses during the evening session (i.e. when homeostatic sleep pressure has accumulated) on SWA in the first sleep cycle [4] and compared these regressions between groups (15 morning and 13 evening types). Whole brain analysis revealed that during the subjective evening hours, BOLD responses in a hypothalamic region (coordinates: −6 −12 −8 mm; Z-value = 3.28; p_svc_ = 0.031) were differently modulated between chronotypes according to SWA at the beginning of the night (Figure 5; see Table S5 for the results of the regression analysis over all chronotypes). The cluster of activation was located more posterior than the anterior hypothalamic area detected in our previous report based on optimal performance measure in the psychomotor vigilance task (PVT; coordinates: 6 −6 −12 mm; [4]). The present area was close to the region reported to show a decrease in grey matter concentration in narcoleptic patients, relative to controls and identified by these authors as the posterior-lateral hypothalamus [22].

Independent regressions within each group showed that interference-related BOLD activity in the posterior part of the hypothalamus was negatively related to SWA at the beginning of the night during the evening hours in morning types (p_svc_ = 0.031), whereas the regression was not significant in evening types.

## Discussion

Our findings indicate that sleep-wake regulation differentially impacts on brain responses to cognitive interference according to chronotype. As expected, at the behavioral level, morning types exhibited higher subjective sleepiness and lower objective vigilance levels than evening types during the evening hours, although the amount of prior wakefulness and circadian phase was the same for both chronotypes in the morning and evening test session. We cannot completely rule out a shorter circadian period in morning relative to evening types [23], but salivary melatonin levels indicated comparable entrainment to the 24-h cycle. In line with previous studies (e.g. [2]), morning types exhibited higher SWA values than evening types during the first NREM sleep episode, despite the same amount of time previously spent awake, suggesting higher levels of accumulated sleep pressure. Together, these data suggest that morning type individuals experience higher vulnerability to increasing time spent awake across a normal waking day. Likewise, morning types tended to perform slower on the Stroop task than evening types. Notwithstanding, the magnitude of the interference effect was not modulated at the behavioral level, since the interaction chronotype by item (i.e. incongruent vs. congruent) did not yield significance. Behavioral interference in the Stroop task has previously been shown resistant to the manipulation of circadian phase and homeostatic sleep pressure (e.g. [24]), confirming the initial hypothesis that the circadian timing system and the sleep-wake homeostat affect cognition depending on the specific cognitive domain. In this perspective, trait-like interindividual differences in vulnerability to increasing time awake have been shown to cluster around three different behavioural dimensions, namely self-evaluation of sleepiness, cognitive processing ability and behavioural alertness, as investigated by sustained attention [25]. The emergence of more than one dimension for objective performance deficits indicates that distinct neurocognitive subsystems may regulate different aspects of the cognitive effects of increasing time spent in the wake state. Our data further indicate that observed changes in regional brain responses can precede behavioural modifications in cognitive interference. Indeed, sleepiness effects secondary to adverse circadian phase or increasing homeostatic pressure may primarily affect pathways downstream from higher order attentional processes [26] such that, decreased alertness probably participates in performance deterioration to the task, which is unlikely to result only from specific effects of sleep loss on higher order control functions [27].

### Chronotype-specific brain responses to cognitive interference throughout a normal waking day

One of our key findings is the significant influence of chronotype and time-of-day on brain responses to cognitive interference. Indeed, responses were maintained or even increased in evening types from morning to evening sessions in a set of brain areas known to be involved in Stroop performance, whereas they decreased in morning types in the same areas and under the same conditions. The identified brain areas have been previously reported to play a pivotal role in successful inhibitory functioning, particularly the anterior cingulate cortex, involved in monitoring and resolution of cognitive conflict [20], [28]. Likewise, the insula has been associated with error processing [21]. In the present study, ceiling effects in accuracy measures during performance on the Stroop task, commonly reported in healthy young participants (e.g. [29]), prevented an analysis of changes in error-related cerebral activity. However, besides its putative implication in error processing, insular activity has been shown to play a generic role in conflict processing (Wager et al., 2005). Also, responses related to cognitive inhibition in the insula area has been previously found to increase in subjects with low vulnerability to sleep deprivation, and to decrease in subjects more vulnerable to sleep loss [30]. In this latter study, different time courses in cortical task-related BOLD responses during task performance differentiated more resilient individuals from those participants more susceptible to the detrimental effects of sleep loss. Our results suggest that a differential impact of sleep pressure on hypothalamic wake promotion may be one of the systems underlying those inter-individual differences in the maintenance of cortical cognition-related cerebral activity.

### Hypothalamic integration of wake promoting signals for the consolidation of cognitive interference in the subjective evening hours

Another key finding of our study is the identification of differential interference-related responses to accumulated sleep pressure between morning and evening types in a region located in the posterior hypothalamus. This relationship suggests that with accumulated homeostatic sleep pressure, present during the evening hours, morning type individuals become progressively less and less apt to recruit this hypothalamic region during Stroop performance. An exact delineation of the specific hypothalamic nuclei responsible for this diminishing response is beyond the current spatial resolution of fMRI. It is worth noticing, that this region is located in a more posterior hypothalamic part than the area we previously observed to be negatively related to accumulated homeostatic sleep pressure during optimal sustained attention performance. The posterior hypothalamic portion contains neurons actively involved in the promotion of wakefulness [31], making this area a potential candidate for modulating brain responses underlying cognitive effort. It is tempting to assume that the arousal stabilizing neuropeptide hypocretin produced in the postero-lateral hypothalamus [32] has some characteristics that suitably fit into the framework of our data. Chief among these is hypocretin expression that follows a circadian variation in rats [33], [34], monkeys [35] and humans [36], but also appears to be regulated by a reactive homeostatic mechanism, opposing to the accumulating sleep drive during the day to maintain wakefulness [35].

In the context of the present study, we surmise that BOLD activity in the posterior hypothalamic region allows the promotion and maintenance of appropriate cognitive interference abilities at the cortical level during the late part of a normal waking day, when homeostatic pressure for sleep is high. The posterior hypothalamic region widely projects to the entire cortex [37], [38] where these projections enhance cortical responses either directly [39] or through projections of the intralaminar thalamus [40]. We suggest a relative disruption in the transmission of similar alerting signals generated by the circadian system into cortical levels in morning types during the evening. Still, the maintenance of preserved behavioral effects also indicates that activity in other brain structures may provide support for the processing of cognitive interference.

### Conclusion

Our results indicate that in the subjective evening hours, conflict processing-related cortical responses are maintained or even enhanced in evening types, whereas they decrease in morning types. We speculate that this effect is driven by changes in the wake-promoting signal generated by hypothalamic structures to ensure sustained alertness and cortical activity despite accumulating sleep pressure. Conversely, in morning chronotypes, the alerting signal may be less pronounced and thus less able to counteract increasing homeostatic sleep pressure in the evening, potentially leading to less stable arousal states and thus challenging cognitive performance and favoring sleep occurrence at earlier times of the day. Along with genetic studies [27], [41], our results shed light on the mechanisms underlying inter-individual differences in behavioral and cerebral responses to changes in circadian and sleep pressure and strengthens chronotype as a strong predictor for such vulnerability.

## Materials and Methods

Supplemental details are presented in *Methods S1*

### Ethics statement

All volunteers fulfilled a written consent form to participate in this study approved by the Ethics Committee of the Faculty of Medicine of the University of Liège, and performed in accordance with the ethical standards described in the Declaration of Helsinki (1964).

### Population

Sixteen extreme morning and 15 extreme evening type subjects gave their written informed consent to participate in this study. All volunteers were healthy and between 22 and 32 years of age (morning types: 9 women/7 men; evening types: 8 women/7 men). Exclusion criteria were reports of medical, psychiatric and sleep disorders, medication or drug use, alcohol abuse, excessive caffeine consumption or physical activity, shift work within the three past months, and transmeridian travel or disturbances in the sleep-wake cycle within one month before the experiment. Subjects were screened according to their timing preferences as defined by the morningness-eveningness questionnaire (MEQ; [12]), in which scores > 70 identify extreme morning types and scores <30 identify extreme evening types. The two groups were matched according to age, sex and educational level (Table 1).

### Procedures

The experimental design is illustrated in Figure 1.

In a first step, participants reported to the sleep facility for a habituation night. After this night, they were requested to follow during one week the sleep schedule (±30 minutes) they would spontaneously adopt while free from any socio-professional constraints while averaging their bedtime to 8 hours (±1 hour). Actimetry (Cambridge Neurotechnologies, UK) assessed participants' compliance to the selected rest-activity patterns. Afterwards, subjects entered the sleep laboratory for 2 subsequent nights (Figure 1). Subjects came to the laboratory 7 hours before habitual lights off on day 1. After the hook-up of electrodes, subjects stayed under controlled conditions in dim light (<10 lux). Subjective sleepiness (Visual analogue scale (VAS) and the Karolinska Sleepiness Scale (KSS; [18]) and objective vigilance (a modified version of the PVT; [19]) were assessed at hourly intervals while awake. Furthermore, hourly collected saliva samples while awake were assayed for melatonin, and polygraphic data were recorded during the nights. After lights off, subjects were allowed to sleep for 8 hours. One and a half (morning session) and 10.5 (evening session) hours after wake up of scheduled sleep timing, subjects underwent an fMRI session during the performance of various cognitive tasks. We report here results related to the Stroop task; results regarding a psychomotor vigilance task are reported elsewhere [4]. For half of the subjects, the morning session followed the first experimental night and the evening session the second one while for the other half of the volunteers, the morning session followed the second experimental night and the evening session the first night, thus controlling for a potential practice effect on session-related Stroop performance. The order of selected cognitive tasks was counterbalanced across subjects and sessions and parallel versions of the Stroop task were used for the repeated administrations.

### Stroop task

The color-word Stroop paradigm [11] investigates the ability to process conflicting information. Participants are required to indicate the color in which a word is printed as quickly as possible while ignoring the word's meaning, an interference occurring when the word is a color name but printed in a different color (e.g. the word “RED” printed in blue). We used a computerized trial by trial, 4 colors version of the task adapted to the fMRI environment. The test comprised 196 items divided into four different categories: congruent (C), incongruent (I) and neutral (NE and NT) trials. Neutral items consisted of a non-verbal stimulus, i.e. a sequence of X's, printed in a particular color. Trials with the same printed color and word meaning were defined as congruent items (e.g. the word RED printed in the color red). By contrast, incongruent trials were represented by color words printed in a different color than the meaning of the word (e.g. RED printed in the color yellow). We used incongruent and congruent items in order to investigate the Stroop interference effect (see Methods S1 for details of the task). Stimuli were presented on a black screen and each color was represented in equal proportions within each trial type. Responses were made by manipulating a 4-buttons response keypad with the dominant hand.

### Polysomnographic recordings

During sleep, EEG was recorded digitally with Brainamp MR Family and V-amp EEG amplifiers (Brain Products Inc. Gilching, Germany), using AgCl electrodes. Recording was done at 250 Hz sampling frequency, with a 0.1 Hz high-pass filter, a 80 Hz low-pass filter, and a 50 Hz notch filter. Electrodes were placed according to the 10–20-System with 4 channels (Fz, Cz, Pz, Oz) referenced to linked mastoids. Additionally, horizontal and vertical eye movements and a submental electromyogram were recorded. Sleep stages during scheduled nights were visually scored on a 20-s epoch basis [42] according to standard criteria [43]. EEG artifacts were visually detected. Finally, NREM–REM sleep cycles were determined according to the criteria of Feinberg & Floyd [44]. Abnormally long first NREM episodes, where evidence of a ‘skipped’ first REM period was observed, were divided into two separate NREM episodes [45].

### Quantitative EEG analysis

EEGs were subjected to spectral analysis (Welch's method, 4-s window, 50% overlap) using a fast Fourier transforms resulting in a 0.5 Hz bin resolution. EEG power spectra were calculated during NREM sleep in the frequency range between 0.5 and 25 Hz. Results were averaged over all epochs of NREM sleep (stage 2–4), which did not contain movement artifacts. The time course of EEG slow wave activity (1–4 Hz) during non-REM sleep was investigated throughout sleep cycles of the nights preceding fMRI acquisitions.

### MRI data acquisition

Functional MRI-series (fMRI) were acquired using a head-only 3T scanner (Siemens *Allegra*). Multislice T2*-weighted fMRI images were obtained with a gradient echo-planar sequence using axial slice orientation (TR = 2130 ms, TE = 40 ms, FA = 90°, 32 transverse slices, 3 mm slice thickness, 30% inter-slice gap, FoV = 220×220 mm^2^, matrix size = 64×64×32, voxel size = 3.4×3.4×3.0 mm^3^). Structural T1-weighted brain images were also acquired [46].

### Functional MRI data analysis

A detailed description of fMRI data analysis can be found in Methods S1.

fMRI data from morning and evening sessions were analyzed using SPM5 (http://www.fil.ion.ucl.ac.uk) implemented in MATLAB 7 (Mathworks, Sherbom, MA). Volumes were corrected for head motion, spatially normalized, and smoothed. Data were processed using two-step statistical analyses, taking into account intra-individual then inter-individual variance, respectively. For each subject, brain responses were modeled at each voxel, using a general linear model. Five separate regressors were included: events associated with congruent trials (C), events related to incongruent trials (I), events associated with neutral items that were used for comparison with other trial conditions (NE), events linked to neutral items used in order to annihilate undesired priming effects (NT), and response errors over all trial types (for those subjects committing errors). Linear contrasts tested the main effect of interference in the Stroop task as revealed by comparing I to C trials (I>C). At the random-effect level, the interference-related effect of chronotype [(I vs. C)*(morning vs. evening types)], of time of day [(I vs. C)*(morning vs. evening session)] and most importantly the interference-related interaction effects between chronotype and time of day [(I vs. C)*(morning vs. evening session)*(morning vs. evening type)] were tested. In a second analysis, a covariate representing the amount of SWA during the first sleep cycle for each group separately was included into the model in order to investigate whether accumulated sleep pressure modulated task-related BOLD responses and whether this effect differed between chronotypes. The resulting set of voxel values constituted a map of *t* statistics [SPM(T)] thresholded at *p*<0.001 (uncorrected; over the whole brain). Statistical inferences were performed after correction for multiple comparisons at a threshold of p<0.05, using Gaussian random field theory at the voxel level in small spherical volumes (radius, 10 mm) around a priori locations of structures of interest, taken from the literature.

## Supporting Information

Figure S1 **Reaction times (±SEM) during the Stroop task according to trial type (congruent [C] and incongruent [I]), testing time (morning versus evening session) and chronotype (blue: evening types; red: morning types).**
(TIF)

Table S1 **Demographic, sleep and circadian characteristics (mean ± SD) in morning and evening types.**
(DOCX)

Table S2 **Visually scored sleep stages (mean ± SD) expressed in percentages according to chronotype (averaged over the two experimental nights).** Comparison between groups: all ps > 0.1).
(DOCX)

Table S3 **Brain regions involved in the Stroop interference effect (Incongruent > Congruent items), all chronotypes and testing sessions confounded.** Reported brain activations are significant after correction for multiple comparisons over the entire volume (*) or over a small volume of interest (svc). R: right hemisphere; L: left hemisphere.
(DOCX)

Table S4 **Task-related main effect of time of day and chronotype.** R: right hemisphere; L: left hemisphere.
(DOCX)

Table S5 **Negative regression between relative SWA in the first sleep cycle (all chronotypes) and BOLD activity involved in the main effect of the Stroop task (I > C trials) during the evening scan session.** p_svc_: significance after small volume correction (radius 10 mm) according to structures of interest reported in the literature. R: right hemisphere; L: left hemisphere. * p<0.001 uncorrected.
(DOCX)

Methods S1 (DOC)
