# Supplementary figures and images for: Circadian Preference Modulates the Neural Substrate of Conflict Processing across the Day
Source: PLoS One. 2012 Jan 4;7(1):e29658. doi: 10.1371/journal.pone.0029658 (PMC3251569; doi:10.1371/journal.pone.0029658)

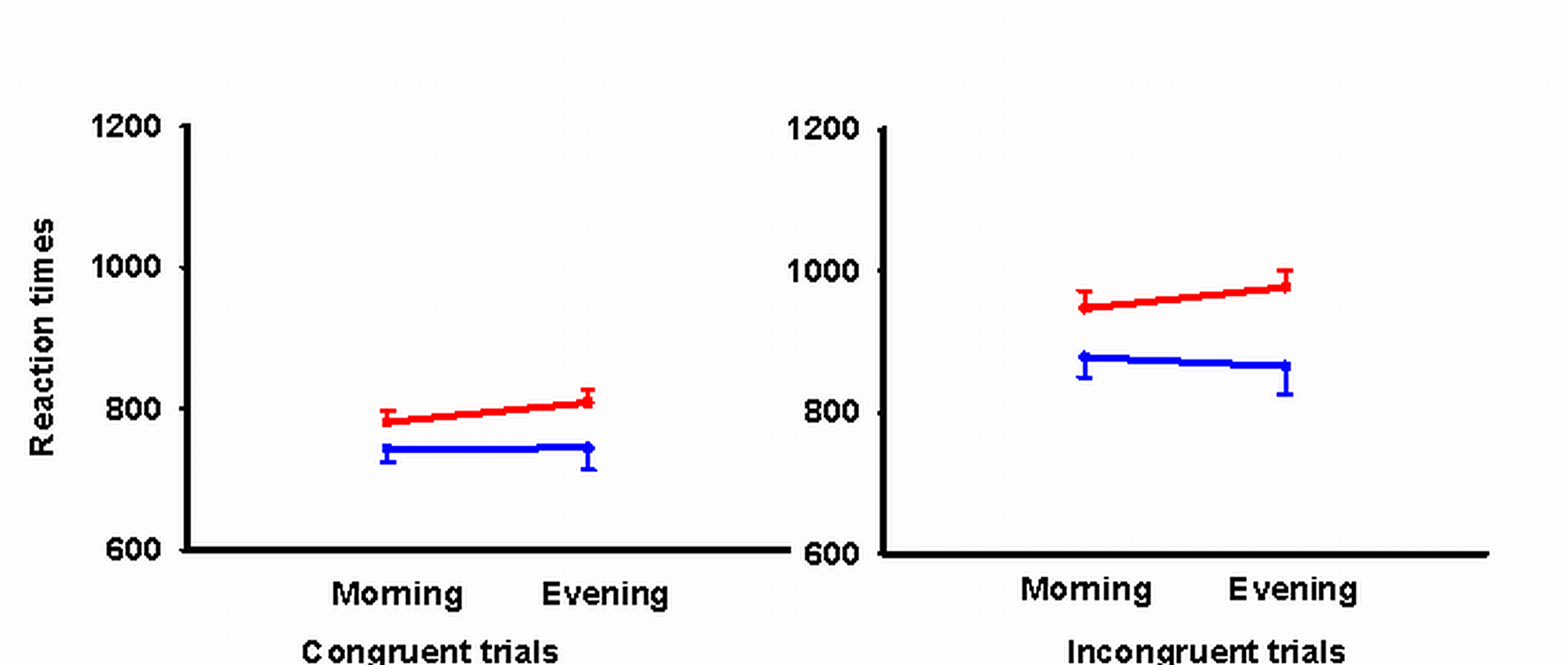

Supplement: Figure S1 — Reaction times (±SEM) during the Stroop task according to trial type (congruent [C] and incongruent [I]), testing time (morning versus evening session) and chronotype (blue: evening types; red: morning types). (TIF) [file pone.0029658.s001.tif]
