# Supplementary material for: Circadian Preference Modulates the Neural Substrate of Conflict Processing across the Day
Source: PLoS One. 2012 Jan 4;7(1):e29658. doi: 10.1371/journal.pone.0029658 (PMC3251569; doi:10.1371/journal.pone.0029658)
Supplement: Table S1 — Demographic, sleep and circadian characteristics (mean ± SD) in morning and evening types. (DOCX) [file pone.0029658.s002.docx]

**Table S1.** Demographic, sleep and circadian characteristics (mean ± SD) in morning and evening types.

|  | **Morning types** | **Evening types** |
| --- | --- | --- |
| Sex | 9w/7m | 8w/7m |
| Age | 24.75 ± 3.9 | 23.5 ± 4.1 |
| Study time | 15,75 ± 3 | 14.98 ± 1.6 |
| Mill Hill | 25.93 ± 4.1 | 24.74 ± 1.4 |
| PSQI | 3.75 ± 1.0 | 4.05 ± 1.03 |
| ESS | 4.56 ± 1.8 | 5.29 ± 3.12 |
| MEQ | 70.30 ± 0.5 | 27.21 ± 1.9 * |
| MCTQ | 6.90 ± 0.4 | 2.91 ± 0.5 * |
| Bedtime | 22:24 ± 8' | 02:50 ± 15' * |
| MRC | 20:21 ± 11' | 24:35 ± 21' * |
| Phase angle | 2:03 ± 8' | 1:42 ± 10' |

**Note.** Mill Hill vocabulary test, with a high loading on crystallized intelligence; PSQI : Pittsburgh Sleep Quality Index; ESS : Epworth sleepiness scale; MEQ : Morningness-Eveningness Questionnaire of Horne and Ostberg ; MCTQ : Munich Chronotype Questionnaire; MRC : Melatonin mid range crossing values. ***** two sample t-test : p < .05
