# Supplementary material for: Circadian Preference Modulates the Neural Substrate of Conflict Processing across the Day
Source: PLoS One. 2012 Jan 4;7(1):e29658. doi: 10.1371/journal.pone.0029658 (PMC3251569; doi:10.1371/journal.pone.0029658)
Supplement: Table S2 — Visually scored sleep stages (mean ± SD) expressed in percentages according to chronotype (averaged over the two experimental nights). Comparison between groups: all ps > 0.1). (DOCX) [file pone.0029658.s003.docx]

**Table S2.** Visually scored sleep stages (mean ± SD) expressed in percentages according to chronotype (averaged over the two experimental nights). Comparison between groups: all ps > 0.1)

|  | **Visual scoring** | | | |  |  |
| --- | --- | --- | --- | --- | --- | --- |
|  | **Morning types** | | **Evening types** | | |  |
| Wake | | 3,18 ± 1.97 | | 3,1 ± 2,74 | | |
| Stage 1 | | 4,53 ± 2.63 | | 3,91 ± 2.49 | | |
| Stage 2 | | 46,64 ± 7.8 | | 45,99 ± 5.8 | | |
| SWS | | 25,54 ± 8.9 | | 24,91 ± 6.4 | | |
| REM | | 23,28 ± 4.0 | | 25,27 ± 3.2 | | |
| Sleep latency (min) | | 8.57 ± 6.94 | | 11.35 ± 5.87 | | |
| TST (min) | | 457.16 ± 14.41 | | 458.46 ± 12.14 | | |
| Efficiency | | 95.1 ± 3.01 | | 95.62 ± 2.53 | | |
