# Supplementary material for: Circadian Preference Modulates the Neural Substrate of Conflict Processing across the Day
Source: PLoS One. 2012 Jan 4;7(1):e29658. doi: 10.1371/journal.pone.0029658 (PMC3251569; doi:10.1371/journal.pone.0029658)
Supplement: Table S3 — Brain regions involved in the Stroop interference effect (Incongruent > Congruent items), all chronotypes and testing sessions confounded. Reported brain activations are significant after correction for multiple comparisons over the entire volume (*) or over a small volume of interest (svc). R: right hemisphere; L: left hemisphere. (DOCX) [file pone.0029658.s004.docx]

| **Table S3**: Brain regions involved in the Stroop interference effect (Incongruent > Congruent items), all chronotypes and testing sessions confounded. Reported brain activations are significant after correction for multiple comparisons over the entire volume (*) or over a small volume of interest (svc). R: right hemisphere; L: left hemisphere | | | | | | | | | |  |  |
| --- | --- | --- | --- | --- | --- | --- | --- | --- | --- | --- | --- |
| ***Brain regions*** | ***Side*** | | ***MNI coordinates*** | | ***Z-score*** | | ***Psvc*** | | ***Coordinates found in*** | | |
| Insula | | R | 36 24 -2 | 5.73 | | 0.000* | |  | | |  |
|  | |  | 36 20 0 | 5.2 | | 0.001 | | [[9](#_ENREF_9)] | | |  |
|  | | L | -36 24 -8 | 5.36 | | 0.002* | |  | | |  |
|  | |  | -32 22 -2 | 4.48 | | 0.001 | | [[9](#_ENREF_9)] | | |  |
| Inferior frontal sulcus | | R | 46 12 30 | 3.8 | | 0.005 | | [[7](#_ENREF_7)] | | |  |
|  | | L | -45 10 28 | 4.58 | | 0.047 | | [[9](#_ENREF_9)] | | |  |
| Intraparietal sulcus | | L | -30 -54 46 | 5.26 | | 0.004* | |  | | |  |
|  | |  | -28 -70 32 | 3.44 | | 0.015 | | [[7](#_ENREF_7)] | | |  |
|  | |  | -26 -72 28 | 3.95 | | 0.003 | | [[7](#_ENREF_7)] | | |  |
| Intraparietal sulcus | | R | 32 -56 46 | 3.66 | | 0.008 | | [[7](#_ENREF_7)] | | |  |
| Anterior cingulate sulcus | | R | 0 18 52 | 4.18 | | 0.001 | | [[7](#_ENREF_7)] | | |  |
|  | |  | 6 20 48 | 3.72 | | 0.006 | | [[7](#_ENREF_7)] | | |  |
|  | |  | 5 28 25 | 3.57 | | 0.012 | | [[10](#_ENREF_10)] | | |  |
|  | | L | -2 22 52 | 3.56 | | 0.011 | | [[7](#_ENREF_7)] | | |  |
|  | |  | -2 28 28 | 3.52 | | 0.012 | | [[9](#_ENREF_9)] | | |  |
|  | |  | -4 26 30 | 3.53 | | 0.012 | | [[10](#_ENREF_10)] | | |  |
| Superior temporal sulcus | | R | 46 -28 -4 | 3.91 | | 0.003 | | [[10](#_ENREF_10)] | | |  |
| Fusiform gyrus | | L | -42 -54 -22 | 3.3 | | 0.022 | | [[10](#_ENREF_10)] | | |  |
