# Supplementary material for: Circadian Preference Modulates the Neural Substrate of Conflict Processing across the Day
Source: PLoS One. 2012 Jan 4;7(1):e29658. doi: 10.1371/journal.pone.0029658 (PMC3251569; doi:10.1371/journal.pone.0029658)
Supplement: Table S4 — Task-related main effect of time of day and chronotype. R: right hemisphere; L: left hemisphere. (DOCX) [file pone.0029658.s005.docx]

**Table S4**. Task-related main effect of time of day and chronotype. R: right hemisphere; L: left hemisphere

| **Higher task-related (I>C) activity in the morning than in the evening session, all chronotypes confounded (p<0.001 uncorrected)** | | | | | | | | | | | | | | |  |  |
| --- | --- | --- | --- | --- | --- | --- | --- | --- | --- | --- | --- | --- | --- | --- | --- | --- |
| ***Brain regions*** | | ***Side*** | | ***MNI coordinates*** | | | ***Z-score*** | | | | | | | | | |
| Middle frontal gyrus | | L | | -36 0 56 | | | 3.70 | | | |  |  |  |  |  |  |
| Inferior frontal gyrus | | L | | -48 6 10 | | | 3.67 | | | |  | |  |  |  |  |
| **Higher task-related (I>C) activity in the evening than in the morning session, all chronotypes confounded** | | | | | | | | | | | | | | | | |
| Postcentral gyrus | | R | | 2 -38 74 | | | 3.13 |  | | | | | | | |  |
| **Higher task-related (I>C) activity in the morning than in the evening chronotypes , all testing sessions confounded (p<0.001 uncorrected)** | | | | | | | | | | | | | | | | |
| Inferior frontal gyrus | | R | | 52 20 -4 | | | 3.27 | | | |  | | | | |  |
| Parahippocampal gyrus | | R | | 30 -28 -14 | | | 3.23 | | | |  | | | | |  |
| Brainstem | | R | | 16 -12 -30 | | | 3.22 | | | |  | | | | |  |
| **Higher task-related (I>C) activity in evening than in morning chronotypes, all testing sessions confounded** | | | | | | | | | | | | | | |  |  |
| Thalamus | | R | | | 8 -28 12 | | 3.24 | |  | | |  | | | |  |
