# Supplementary material for: Circadian Preference Modulates the Neural Substrate of Conflict Processing across the Day
Source: PLoS One. 2012 Jan 4;7(1):e29658. doi: 10.1371/journal.pone.0029658 (PMC3251569; doi:10.1371/journal.pone.0029658)
Supplement: Table S5 — Negative regression between relative SWA in the first sleep cycle (all chronotypes) and BOLD activity involved in the main effect of the Stroop task (I > C trials) during the evening scan session. psvc: significance after small volume correction (radius 10 mm) according to structures of interest reported in the literature. R: right hemisphere; L: left hemisphere. * p<0.001 uncorrected. (DOCX) [file pone.0029658.s006.docx]

**Table S5**: Negative regression between relative SWA in the first sleep cycle (all chronotypes) and BOLD activity involved in the main effect of the Stroop task (I > C trials) during the evening scan session. p_svc_ : significance after small volume correction (radius 10 mm) according to structures of interest reported in the literature. R: right hemisphere; L: left hemisphere. * p < 0.001 uncorrected.

| ***Brain regions*** | ***Side*** | ***MNI coordinates*** | ***Z-score*** | ***Psvc*** | ***Coordinates found in*** |
| --- | --- | --- | --- | --- | --- |
| Middle frontal gyrus | R | 28 42 4 | 3.68 | * |  |
| Anterior hypothalamus | R | 4 2 -4 | 2.77 | 0.061 | [[11](#_ENREF_11)] |
| Brainstem | R | 2-32 -20 | 2.95 | 0.069 | [[5](#_ENREF_5)] |
