# Supplementary material for: Circadian Preference Modulates the Neural Substrate of Conflict Processing across the Day
Source: PLoS One. 2012 Jan 4;7(1):e29658. doi: 10.1371/journal.pone.0029658 (PMC3251569; doi:10.1371/journal.pone.0029658)
Supplement: Methods S1 — (DOC) [file pone.0029658.s007.doc]

**Methods S1**

**Supplemental Methods**

*Procedures*

Individual sleep schedules were determined according to individual preferred sleep and wake timing. Criteria for such timing preferences included sleep schedules adopted on free days as assessed by the Munich Chronotype Questionnaire and after interviewing the participant to ensure that the scheduled timing was as close as possible to the schedule that he or she would spontaneously adopt. In a second step, the successfully screened participants reported to the sleep facility for a habituation night. After this night, they were asked to follow the sleep schedule (± 30 minutes) they would spontaneously adopt while free of any socio-professional timing constraints but adhering to a bedtime of 8 hours (± 1 hour). To assess participants’ compliance to the selected rest-activity patterns, motor activity of the non-dominant arm was recorded with actimeters (Cambridge Neurotechnologies, UK) during the week prior the experimental sessions along with sleep-wake logs. After one week of actimetry recording, the volunteers entered the sleep laboratory for 2 subsequent nights (Figure 1).

The precise schedule of each session was individually adapted according to the subject’s habitual bedtime based on the mean timing of its sleep midpoint derived from the actimetry data of the preceding week. Study participants reported 7 hours before habitual lights off to the laboratory on day 1. After the hook-up of the electrodes they lived under constant conditions in dim light (< 10 lux). Subjective sleepiness (Visual analogue scale (VAS) and the Karolinska Sleepiness Scale (KSS; ) and objective vigilance (a modified version of the PVT; ) were assessed at hourly intervals while awake. Furthermore, hourly collected saliva samples were assayed for melatonin using a direct double-antibody radioimmunoassay validated by gas chromatography-mass spectroscopy with an analytical least detectable dose of 0.65 pg/ml (Bühlmann Laboratories, Switzerland; ). Polygraphic data (see below) were recorded during the night. After lights off, subjects were allowed to sleep for 8 hours. One and a half (subjective morning session) and 10.5 (subjective evening session) hours after wake up of scheduled sleep timing, subjects underwent an fMRI session during the performance of various cognitive tasks. We report here results related to the Stroop task. Results of a psychomotor vigilance task are reported elsewhere . For half of the subjects, the morning session followed the first experimental night and the evening session the second one while for the other half of the volunteers, the morning session followed the second experimental night and the evening session the first night, thus controlling for a potential practice effect on session-related Stroop performance. Subject stayed in a dimly lit (<10 lux) room for at least 4 hours before the scanning sessions. The order of selected cognitive tasks was counterbalanced across subjects and sessions.

*Stroop task*

The color-word Stroop paradigm consists in a classical interference task aiming at investigating the ability to process conflicting information. This requires the subjects to suppress the tendency to make an automatic response in favor of an alternative stimulus-response mapping . In this task, subjects are required to indicate the color in which a word is printed as quickly as possible while ignoring the word’s meaning, an interference occurring when the word is a color name but printed in a different color (e.g. the word “RED” printed in blue). We used a computerized trial by trial, 4 colors version of the task adapted to the fMRI environment using Cogent2000 (http://www.vislab.ucl.ac.uk/cogent.php) implemented in MATLAB 6.1 (Mathworks Inc., Sherbom, MA). The test comprised 196 items divided into four different categories: congruent (C), incongruent (I) and neutral (NE and NT) trials. Trials with the same printed color and word meaning were defined as congruent items (n = 49; e.g. the word RED printed in the color red). By contrast, incongruent trials (n = 49) were represented by color words printed in a different color than the meaning of the word (e.g. RED printed in the color yellow). Finally, neutral items consisted of a non-verbal stimulus, i.e. a sequence of X’s, printed in a particular color. Half of the neutral trials (NE = 48) consisted in “real” neutral events and were used in the statistical analysis. The other half of the neutral trials (NT = 50) systematically followed incongruent trials and were printed in a colour which was neither the colour of the word nor the printed colour of the subsequent item. The latter were placed in order to annihilate non desired priming effects on the incongruent and congruent items used for analysis purposes. A further reason to selectively target on congruent and incongruent trials was that, in contrast to the neutral trials we used, these items consisted in verbal semantically meaningful material, thus more suitable to the identification of the cerebral correlates underlying cognitive interference per se.

The stimuli were presented on a black screen and each color was represented in equal proportions within each trial type. The items remained on the screen until an answer was provided. After the response, the stimulus was replaced by a white fixation cross at the centre of the black screen. The duration of the fixation cross was 2 seconds, except in approximately 8% of the cases, where the fixation cross stayed for 10 seconds in order to add a further jitter to the task. Responses were made by manipulating a 4-buttons response keypad with the dominant hand. Response accuracy and reaction times (RTs) were recorded according to the trial type.

*Statistical analysis of behavioral and electrophysiological measures*

Two-sample t-tests were used to compare the two groups for sleep and wake times, and circadian phase (mid range crossing (MRC) values and phase angles). EEG spectral activity in the SWA band was analyzed across the first four NREM–REM sleep cycles via repeated measure ANOVA on absolute SWA values (averaged over the two experimental nights; except for 2 subjects where only 1 night was available). The first four sleep cycles were used because it was the minimum number of sleep cycles observed in all of the subjects. For the regression analysis with task-related BOLD activity, SWA during the first sleep cycle relative to average SWA across the 4 sleep cycles was used (see ). Time of day and chronotype modulations in Stroop performance were assessed using repeated measure ANOVA on reaction time measures including the within factors “trial type” and “testing time” and the group factor “chronotype”. In case of significant interaction profiles, post-hoc (two sample t-tests) analyses were performed. Differences in subjective sleepiness values, as assessed by ordinal values obtained on the Karolinska Sleepiness Scale before the evening and morning scan sessions were investigated using non-parametric Mann-Withney U tests.

*Functional MRI data acquisition and analysis*

Functional MRI-series (fMRI) were acquired using a head-only 3T scanner (Siemens, *Allegra*, Erlangen, Germany). Multislice T2*-weighted fMRI images were obtained with a gradient echo-planar sequence using axial slice orientation (TR = 2130 ms, TE = 40 ms, FA = 90°, 32 transverse slices, 3 mm slice thickness, 30% inter-slice gap, FoV = 220×220 mm², matrix size = 64×64×32, voxel size = 3.4×3.4×3.0 mm³). The three initial scans were discarded to avoid T1 saturation effects. For anatomical reference, a high-resolution T1-weighted image was acquired (3D MDEFT ; repetition time = 7.92ms, echo time = 2.4ms, inversion time = 910ms, flip angle = 15°, field of view 256 x 224 mm², matrix size = 256 x 224 x 176, voxel size = 1x1x1 mm³). fMRI data from morning and evening sessions were analyzed using SPM5 (http://www.fil.ion.ucl.ac.uk) implemented in MATLAB 7 (Mathworks, Sherbom, MA). Functional scans were realigned using iterative rigid body transformations that minimize the residual sum of square between the first and subsequent images. They were normalized to the Montreal Neurological Institute (MNI) EPI template (two-dimensional spline; voxel size, 2 x 2 x 2) and spatially smoothed with a Gaussian kernel with full width at half-maximum (FWHM) of 8 mm. Data were processed using two-step analyses, taking into account intra-individual then inter-individual variance, respectively. For each subject, brain responses were modeled at each voxel, using a general linear model. Five separate regressors were entered into the model : events associated with congruent trials (C), events related to incongruent trials (I), events associated with neutral items that were used for comparison with other trial conditions (NE), events linked to neutral items used in order to annihilate undesired priming effects (NT), and response errors over all trial types (for those individuals who committed errors: 10 out of 16 morning types and 12 out of 15 evening types during the morning session; 13 morning and 11 evening types during the evening session). The analysis of fMRI data focused on the contrast between congruent and incongruent trials. This decision was made for a couple of reasons. Our task was constructed in such a way that congruent and incongruent trials were always separated by one of the two neutral trial types in order to ensure that the processes underlying congruent and incongruent trials were not interfering with each other. Also, the neutral trials we used consisted in non-verbal stimuli (i.e. a sequence of X’s). The comparison of such non-semantic material to colours words having a semantic meaning would have been confusing for the identification of the cerebral correlates underlying cognitive interference per se.

For each trial type, each single event was modeled as a function representing its onset. The ensuing vector was convolved with the canonical hemodynamic response function, and used as a regressor in the individual design matrix. Movement parameters estimated during realignment (translations in *x*, *y*, and *z* directions and rotations around *x*, *y*, and *z* axes) and a constant vector were also included in the matrix as a variable of no interest. Linear contrasts estimated the main effect of task performance (I > C) at the different testing sessions. High-pass filter was implemented using a cutoff period of 128 s to remove low-frequency drifts from the time series. Serial autocorrelations were estimated with a restricted maximum likelihood algorithm using an autoregressive model of order 1 (white noise). The resulting set of voxel values constituted a map of *t* statistics [SPM(T)]. The individual summary statistical images were spatially smoothed with a Gaussian kernel of 6 mm FWHM and used in a second-level analysis, corresponding to a random-effects analysis. The second-level analysis consisted of a two-sample *t* test contrasting the two groups of subjects and including a covariate representing the amount of SWA during the first sleep cycle for each group separately, centered over the group mean.

The resulting set of voxel values was thresholded at *p* < 0.001 (uncorrected). At the group level, the contrasts of interest consisted in the main effects of the Stroop task as revealed by comparing I to C trials (I > C); the interference-related main effect of chronotypes (I vs C)*(morning vs evening types); interference-related main effects of time of day (I vs C)*(morning vs evening session) and most importantly interference-related interaction effects between chronotype and time of day (I vs C)*(morning vs evening session)*(morning vs evening types). For the relationship between task-related BOLD activity and SWA at the beginning of the night, we were interested in comparing this relation between chronotypes during the evening session since the amount of SWA at the beginning of the night represents a reliable indicator of homeostatic sleep pressure built up accumulated throughout the day. At the second level analysis, statistical inferences were corrected for multiple comparisons using Gaussian random field theory at the voxel level in small spherical volumes (radius, 10 mm) around a priori locations of structures of interest, taken from the literature.

**Supplemental References.**

1. Roenneberg T, Wirz-Justice A, Merrow M (2003) Life between clocks: daily temporal patterns of human chronotypes. J Biol Rhythms 18: 80-90.

2. Akerstedt T, Gillberg M (1990) Subjective and objective sleepiness in the active individual. Int J Neurosci 52: 29-37.

3. Dinges DF, & Powell, J. W. (1985) Microcomputer analyses of performance on a portable, simple visual RT task during sustained operations. Behavior Research Methods, Instruments, &

Computers: A Journal of the Psychonomic Society, 17: 625–655.

4. Weber JM, Schwander, J:C:, Unger, I., Meier, D. (1997) A direct ultrasensitive RIA for the determination of melatonin in human saliva : comparison with serum levels. Journal of Sleep Research 26: 757.

5. Schmidt C, Collette F, Leclercq Y, Sterpenich V, Vandewalle G, et al. (2009) Homeostatic sleep pressure and responses to sustained attention in the suprachiasmatic area. Science 324: 516-519.

6. Stroop JR (1935 ) Studies of interference in serial verbal reactions. Journal of Experimental Psychology 18 643-662.

7. Roberts KL, Hall DA (2008) Examining a supramodal network for conflict processing: a systematic review and novel functional magnetic resonance imaging data for related visual and auditory stroop tasks. J Cogn Neurosci 20: 1063-1078.

8. Deichmann R, Schwarzbauer C, Turner R (2004) Optimisation of the 3D MDEFT sequence for anatomical brain imaging: technical implications at 1.5 and 3 T. Neuroimage 21: 757-767.
